# A Giant Mesenteric Cystic Lymphangioma Causing Abdominal Mass Effect: A Case Report of a 57‐Year‐Old Woman

**DOI:** 10.1155/cris/6450259

**Published:** 2026-05-09

**Authors:** Tesfaye Birhanu Abebe, Daba Iticha Ayana, Ayana Guto Bone, Kirubel Adrissie Barkneh, Bezawit Dereje Tilahun

**Affiliations:** ^1^ School of Medicine, College of Health Science, Salale University, Fitche, Ethiopia, slu.edu.et; ^2^ Department of Surgery, School of Medicine, Collage of Health Science, Salale University, Fitche, Ethiopia, slu.edu.et; ^3^ Department of Pathology, School of Medicine, Collage of Health Science, Salale University, Fitche, Ethiopia, slu.edu.et; ^4^ Ethio-Istanbul General Hospital, Addis Ababa, Ethiopia; ^5^ School of Medicine, College of Medicine and Health Science, University of Gondar, Gondar, Ethiopia, uog.edu.et

**Keywords:** case report, giant cyst, mass effect, mesenteric cyst, surgery

## Abstract

Mesenteric cystic lymphangioma (MCL) is a rare benign abdominal tumor. This report describes a giant MCL in a 57‐year‐old woman presenting with progressive abdominal distension and dyspnea. Examination revealed a massive abdominal mass. Magnetic resonance imaging (MRI) showed a giant cyst (34.8 cm × 46.5 cm × 25.0 cm) with T2 hyperintensity and T1 hypointensity. Laparotomy revealed a 46 cm cyst arising from the ileal mesentery. Complete en bloc excision was performed. Histopathology confirmed MCL with endothelial‐lined channels and lymphoid aggregates; the cyst contained greenish fluid. The patient recovered well with symptom resolution. MCL should be considered in the differential diagnosis of massive abdominal cysts, even in adults. Complete surgical excision remains the definitive treatment, offering excellent outcomes when performed adequately. This case highlights how delayed diagnosis due to healthcare access limitations can lead to extreme presentations requiring complex management.

## 1. Introduction

Mesenteric cysts are rare benign intra‐abdominal lesions with an estimated incidence of 1 in 100,000–250,000 hospital admissions [1], representing a heterogeneous group classified by their tissue of origin—lymphatic, mesothelial, enteric, urogenital, teratomatous, or pseudocysts—according to the Ros‐De Perrot system [2]. Among these, mesenteric cystic lymphangioma (MCL) constitutes a distinct subtype of lymphatic origin. While ~40%–45% of mesenteric cysts remain asymptomatic, larger lesions typically present with abdominal distension or nonspecific complaints. Giant variants exceeding 10 cm in diameter may cause compressive symptoms [3], most commonly gastrointestinal disturbances, though respiratory compromise due to diaphragmatic impingement—as seen in our case—represents an exceptionally rare but significant complication. The diagnostic pathway relies heavily on imaging characteristics: ultrasound typically reveals anechoic cystic structures with posterior acoustic enhancement, while magnetic resonance imaging (MRI) demonstrates pathognomonic T2 hyperintensity and T1 hypointensity [4]. However, histopathological examination remains definitive for confirming the diagnosis and excluding malignant variants. This report documents a 34.8 cm × 46.5 cm × 25.0 cm MCL that developed over 8 months in a 57‐year‐old woman from a rural setting. To our knowledge, this represents one of the largest MCLs documented in the literature, exceeding previously reported cases in overall volume [5].

## 2. Case Presentation

A 57‐year‐old woman presented with an 8‐month history of progressive abdominal swelling that began in the periumbilical region. Initially, she experienced bloating, persistent abdominal distension, and intermittent digestive discomfort. Over the past 2 months, the swelling enlarged rapidly and became associated with significant abdominal pain. She also developed new‐onset shortness of breath, which severely limited her daily activities. She had no prior history of abdominopelvic surgery, nor any personal or family history of similar illness, diabetes mellitus, or hypertension. On physical examination, the patient appeared acutely ill and in visible distress. Vital signs were normal except for tachypnea (RR: 24/min) and respiratory distress, though oxygen saturation remained stable. Abdominal examination revealed gross distension with flank fullness and a firm, nontender intra‐abdominal mass measuring 48 cm in its largest diameter, confined to the abdomen without pelvic extension. Other systems were unremarkable.

Laboratory investigations, including complete blood count, pregnancy test, and organ function tests, were normal. Ultrasound revealed a 32.5 cm × 46.3 cm anechoic cystic structure with thin walls and posterior acoustic enhancement, but no solid components. Subsequent MRI confirmed a 34.8 cm × 46.5 cm × 25 cm mesenteric cyst with characteristic T2 hyperintensity and T1 hypointensity, displacing bowel loops posterolaterally (Figure 1).

Figure 1Preoperative MRI. (A) Coronal and (B) sagittal T2‐weighted images show a well‐defined 34.8 cm × 46.5 cm × 25.0 cm mesenteric cyst with characteristic T2 hyperintensity and T1 hypointensity. Significant mass effect is evident, with posterolateral displacement of bowel loops and marked right hemidiaphragm elevation.

**Figure:**
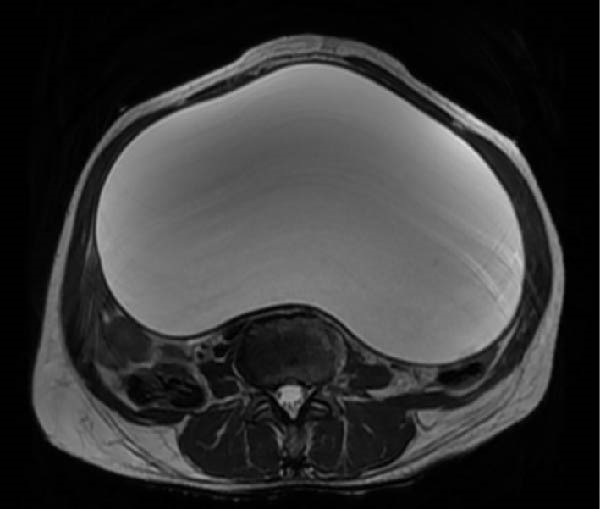
(A)

**Figure:**
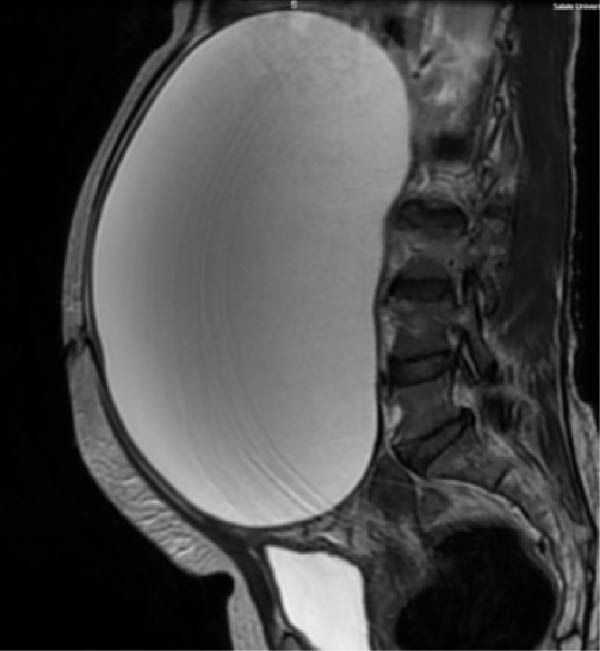
(B)

At laparotomy through a midline incision, a 35 cm × 46 cm cystic mass arising from the ileal mesentery was found (Figure 2), with adhesions to the uterus but normal adnexa. The mass was completely excised without rupture.

Figure 2Intraoperative images. (A) Midline incision exposing the cyst’s smooth, shiny surface. (B) The 35 cm × 46 cm cyst delivered from the abdomen, demonstrating its narrow attachment to the ileal mesentery.

**Figure:**
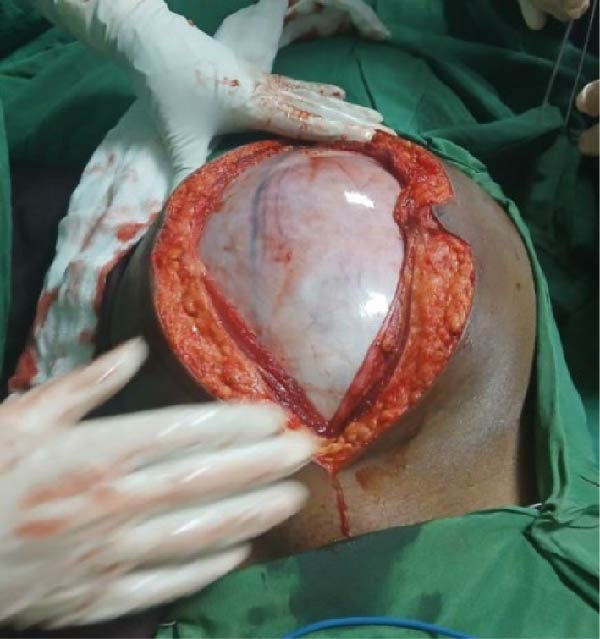
(A)

**Figure:**
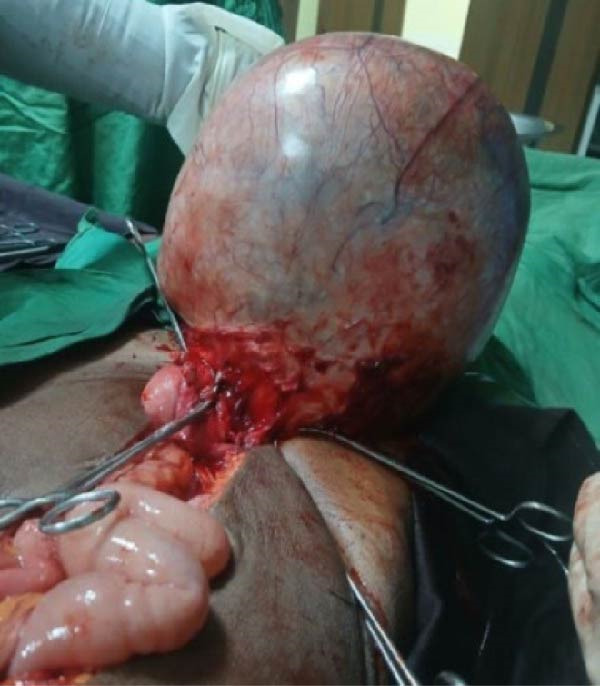
(B)

Histopathologic examination of the resected specimen revealed a smooth, thin‐walled cystic mass. The cut surface was unilocular and contained greenish fluid. Microscopic analysis with hematoxylin and eosin (H&E) staining confirmed the diagnosis of MCL, demonstrating characteristic thin‐walled lymphatic channels lined by flattened endothelial cells within a fibrous stroma containing smooth muscle bundles and lymphoid aggregates (Figure 3). No features of malignancy were identified. The patient recovered well and was discharged on postoperative day 3. At her 6‐month follow‐up, she showed complete resolution of all symptoms with no evidence of recurrence on clinical examination.

Figure 3Histopathologic features of mesenteric cystic lymphangioma. (A) 10x magnification reveals thin‐walled, dilated lymphatic channels lined by flattened endothelial cells within a fibrous stroma. (B) 100x magnification shows lymphoid aggregates and smooth.

**Figure:**
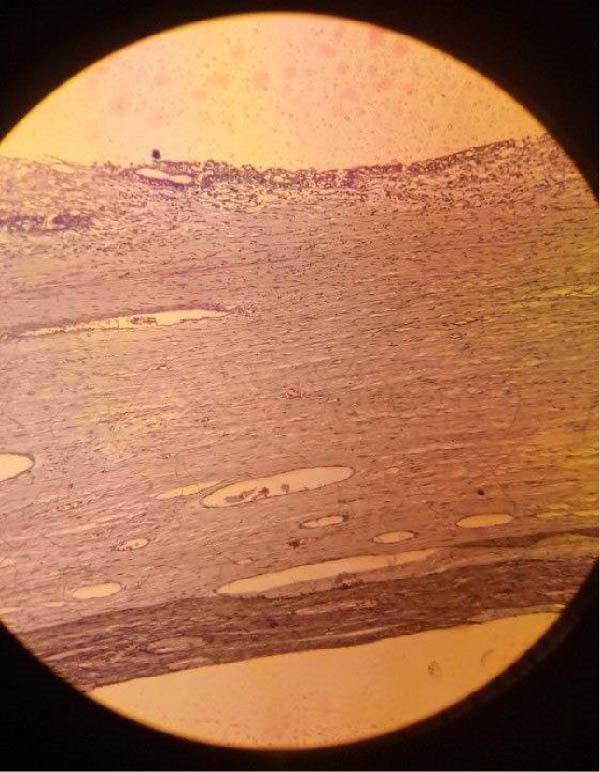
(A)

**Figure:**
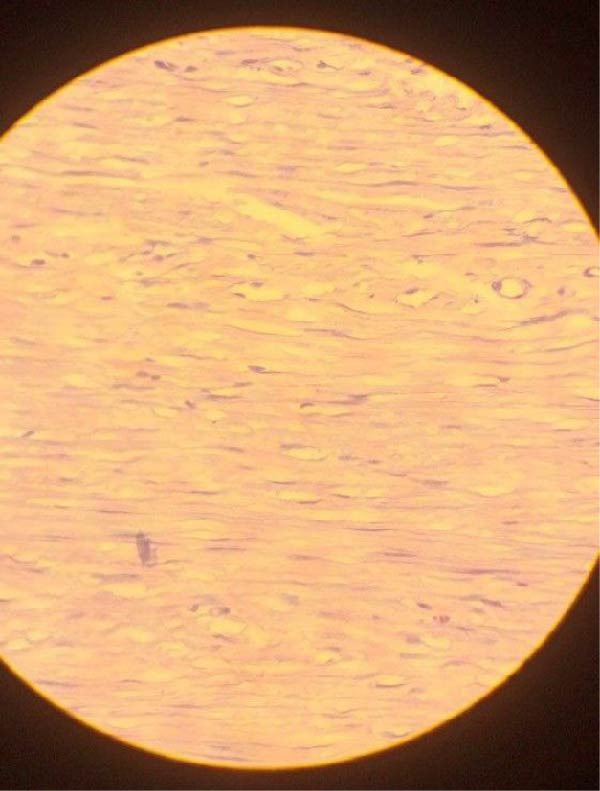
(B)

## 3. Discussion

MCL is a rare benign tumor of lymphatic origin, with reported incidence varying by clinical setting. Among general hospital admissions, the incidence ranges from 1 per 20,000 to 250,000 [6–9]. In contrast, among adult surgical admissions, the estimated incidence is higher at 1 per 20,000 to 50,000 [10], reflecting the selected nature of surgical populations. Even higher rates of ~1 per 20,000 have been reported in pediatric series [10, 11]. According to the Ros‐De Perrot classification system [2, 12], mesenteric cysts are categorized by tissue of origin into six distinct types: (a) lymphatic (including simple lymphangiomas and MCLs), (b) mesothelial (encompassing both benign and malignant cystic mesotheliomas), (c) enteric, (d) urogenital, (e) mature cystic teratomas, and (f) nonpancreatic pseudocysts. Among these, only malignant cystic mesothelioma carries malignant potential, sometimes mimicking benign variants grossly and requiring careful histological differentiation [13]. Lymphangiomas most commonly occur in the cervical region (75%), followed by the axillary region (20%) [14, 15]. The remaining 5% involve rare sites, including the mediastinum (1%) [14], retroperitoneum, peritoneal cavity, bones, and extremities. The incidence of lymphangiomas in the abdominal cavity is less than 5% [16], with peritoneal involvement being exceptionally rare—accounting for only a fraction of these cases and making our presentation particularly unusual. Histologically, these lesions are characterized by thin‐walled cysts with multiple septations composed of endothelial cells, smooth muscle fibers, foam cells, and lymphoid tissue [17]. Mesenteric cyst lymphangiomas represent uncommon abdominal pathologies with diverse and often unclear origins [18]. Current hypotheses indicate these lesions may develop from malformations of lymphatic tissues within the mesentery or following traumatic injury [19, 20]. Anatomically, the majority (66%) are found in the small intestinal mesentery, while approximately one‐third occur in colonic mesentery (primarily affecting ascending and transverse portions) [21]. Rarely (under 1%), these cysts develop in distal colonic regions, including the descending colon, sigmoid, or rectal areas [19]. The condition predominantly affects middle‐aged adults, with peak incidence during the fifth decade [21], and frequently remains clinically silent. Symptomatic presentations, when they occur, typically involve vague abdominal manifestations such as bloating, distension, or digestive discomfort [22]. A systematic review of the literature confirms that our case (34.8 cm × 46.5 cm × 25.0 cm) represents one of the largest adult MCLs reported to date, with giant variants exceeding 20 cm being exceptionally rare. While Parker et al. [5] reported a 42 cm × 47 cm × 10 cm MCL—the largest by two‐dimensional measurement—that case was discovered incidentally in an asymptomatic young man and required small bowel resection due to vascular involvement. Murakami et al.[4] documented a 20 cm jejunal mesenteric lymphangioma in a 26‐year‐old man that was successfully excised without bowel resection by aspirating cyst contents to facilitate dissection from the duodenum. Losanoff et al.[8] reported a 30 cm × 12 cm × 10 cm MCL, and another case measured 14.9 cm × 12.8 cm × 14.9 cm [23]. A 16 cm × 7 cm × 5 cm jejunal mesenteric lesion has also been described [24]. In the largest published series, Aprea et al.[25] documented five adult MCL cases ranging from 3.5 to 18 cm. In comparison, our case demonstrates substantially larger overall volume, contributing to the unique clinical presentation of tachypnea and shortness of breath due to diaphragmatic impingement—a finding absent in all prior reports. Furthermore, consistent with observations from other low‐resource settings [1], our case highlights how delayed presentation in rural populations can lead to extraordinary cyst growth while still permitting successful complete excision without bowel resection. Mesenteric cysts typically present with characteristic imaging features on ultrasound and MRI. On ultrasound examination, these lesions appear as large anechoic cystic structures with thin walls and posterior acoustic enhancement [26]. MRI serves as the gold standard for characterizing abdominal cystic lesions, with lymphangiomas demonstrating pathognomonic features: marked T2 hyperintensity, T1 hypointensity (unless hemorrhagic), and a characteristic multilocular “honeycomb” architecture with numerous delicate septations [27]. These lesions lack solid components or contrast enhancement. In contrast, *benign cystic mesothelioma* (BCM), while also T2‐hyperintense, typically presents as a unilocular or paucilocular cystic mass with thicker, more irregular septations and occasional mild peripheral enhancement [28]. BCMs frequently show peritoneal‐based distribution (unlike lymphangiomas’ mesenteric predilection) and may demonstrate subtle contrast uptake along septa or walls [29]. Neither lesion exhibits malignant features (e.g., nodularity or infiltrative growth), but BCMs have higher recurrence rates post‐resection. Hemorrhage or proteinaceous content can modify signal intensity in both entities, though lymphangiomas more commonly exhibit fluid–fluid levels due to lymphatic fluid dynamics. The patient underwent complete surgical excision via laparotomy, which remains the gold standard treatment for MCLs [7]. MCLs of this magnitude present specific technical challenges, including limited abdominal working space, dense adhesions to surrounding structures (such as the uterine adhesions encountered in our case), risk of intraoperative rupture with subsequent chemical peritonitis, potential involvement of mesenteric vasculature requiring careful dissection to preserve bowel viability, and difficulty achieving complete exposure through standard incisions. These challenges necessitated an open surgical approach, as laparoscopic excision—while preferred for smaller lesions—was impractical given the cyst’s enormous size and the lack of appropriate equipment in our resource‐limited setting. Despite these obstacles, we achieved complete en bloc resection without cyst rupture or intestinal resection, an outcome that compares favorably to some smaller MCLs that required organ resection due to vascular involvement [30] ‐ would have been impractical and actually, the setup was not available. The surgical principles adhered to established guidelines: complete en bloc resection was achieved without cyst rupture, adjacent organ preservation was prioritized where possible, and marsupialization was avoided due to its high recurrence [8, 31].

This case demonstrates that MCLs, though benign, may reach extraordinary sizes with delayed diagnosis. Even giant lesions remain surgically curable when managed properly. MRI is indispensable for preoperative planning, and the patient’s prolonged symptomatology reflects healthcare access disparities in rural populations [1]. The successful outcome in this case—with complete symptom resolution and no recurrence at 6‐month follow‐up—reinforces current management paradigms for MCLs, which are generally associated with excellent prognosis and a low recurrence rate ranging from 0% to 13.6% [32].

## Author Contributions

Tesfaye Birhanu Abebe conceptualized, wrote the original draft of the manuscript, reviewed, edited, and visualized the final manuscript. Daba Iticha Ayana managed the patient, conceptualized, wrote the original draft of the case presentation, assisted in manuscript revision and data curation. Ayana Guto Bone reviewed and edited the manuscript and supervised. Kirubel Adrissie Barkneh and Bezawit Dereje Tilahun assisted in manuscript revision and data curation.

## Funding

No funding was received.

## Disclosure

All authors read and approved the final manuscript.

## Ethics Statement

Approval for this case report was not required to secure from the Ethics Committee of the institution.

## Consent

Written informed consent was obtained from the patient for publication and any accompanying images. A copy of the written consent is available for review by the Editor‐in‐Chief of this journal on request.

## Conflicts of Interest

The authors declare no conflicts of interest.

## Data Availability

The data supporting the findings of this case report are available from the corresponding author upon reasonable request. The journal may review the underlying data to verify the findings if necessary.
